# The Role of Strigolactone in the Cross-Talk Between *Arabidopsis thaliana* and the Endophytic Fungus *Mucor* sp.

**DOI:** 10.3389/fmicb.2018.00441

**Published:** 2018-03-19

**Authors:** Piotr Rozpądek, Agnieszka M. Domka, Michał Nosek, Rafał Ważny, Roman J. Jędrzejczyk, Monika Wiciarz, Katarzyna Turnau

**Affiliations:** ^1^Małopolska Centre of Biotechnology, Jagiellonian University, Kraków, Poland; ^2^Institute of Environmental Sciences, Jagiellonian University, Kraków, Poland; ^3^Institute of Biology, Pedagogical University of Kraków, Kraków, Poland; ^4^Faculty of Biochemistry, Biophysics and Biotechnology, Jagiellonian University, Kraków, Poland

**Keywords:** strigolactone, fungal endophytes, *Arabidopsis thaliana*, salicylic acid, symbiosis, jasmonic acid

## Abstract

Over the last years the role of fungal endophytes in plant biology has been extensively studied. A number of species were shown to positively affect plant growth and fitness, thus attempts have been made to utilize these microorganisms in agriculture and phytoremediation. Plant-fungi symbiosis requires multiple metabolic adjustments of both of the interacting organisms. The mechanisms of these adaptations are mostly unknown, however, plant hormones seem to play a central role in this process. The plant hormone strigolactone (SL) was previously shown to activate hyphae branching of mycorrhizal fungi and to negatively affect pathogenic fungi growth. Its role in the plant–endophytic fungi interaction is unknown. The effect of the synthetic SL analog GR24 on the endophytic fungi *Mucor* sp. growth, respiration, H_2_O_2_ production and the activity of antioxidant enzymes was evaluated. We found fungi colony growth rate was decreased in a GR24 concentration dependent manner. Additionally, the fungi accumulated more H_2_O_2_ what was accompanied by an altered activity of antioxidant enzymes. Symbiosis with *Mucor* sp. positively affected *Arabidopsis thaliana* growth, but SL was necessary for the establishment of the beneficial interaction. *A. thaliana* biosynthesis mutants *max1* and *max4*, but not the SL signaling mutant *max2* did not develop the beneficial phenotype. The negative growth response was correlated with alterations in SA homeostasis and a significant upregulation of genes encoding selected plant defensins. The fungi were also shown to be able to decompose SL *in planta* and to downregulate the expression of SL biosynthesis genes. Additionally, we have shown that GR24 treatment with a dose of 1 μM activates the production of SA in *A. thaliana*. The results presented here provide evidence for a role of SL in the plant–endophyte cross-talk during the mutualistic interaction between *Arabidopsis thaliana* and *Mucor* sp.

## Introduction

A growing number of evidence indicates that endophytic fungi play a significant role in plant biology (Schulz, 2006; Yuan et al., 2009; Johnson et al., 2013). Endophytic fungi facilitate water and nutrient acquisition, resistance to abiotic stress such as drought, salinity and metal stress and provide protection against pathogenic microorganisms and herbivores. Their popularity is growing, for their potential in agriculture and bioremediation (Oelmüller et al., 2009; Li et al., 2012; Johnson et al., 2013). The potential for application of beneficial fungi seems very optimistic, however, understanding the mechanisms of the interactions between plants and endophytic fungi requires extensive research.

Plant adaptation to biotic and abiotic constraints requires several adjustments in plant metabolism, morphology, life cycle *etc*. These adaptations are often mediated by phytohormones. Recently, the plant hormone strigolactone (SL) has been recognized due to its role in root and shoot architecture determination and plant interactions in the rhizosphere (Rochange, 2010; Foo and Reid, 2013; Koltai and Kapulnik, 2013; Pandya-Kumar et al., 2014; Chen et al., 2015; van Zeijl et al., 2015). SLs are carotenoid derivates synthesized from β-carotene by consecutive action of a β-carotene isomerase, two carotenoid cleavage dioxygenases: CCD7 and CCD8 (MORE AXILARY GROWTH-MAX3 and 4) respectively and an enzyme from the P450 cytochrome family: MAX1 (MORE AXILARY GROWTH1). Downstream, a LATERAL BRANCHING OXYREDUCTASE (LBO) was recently shown to convert a carlactone intermediate in the process of SL biosynthesis (Brewer et al., 2016).

Strigolactones are important in plant responses to nutrient and water deficiency (López-Ráez et al., 2008; Yoneyama et al., 2012; Foo et al., 2013; López-Ráez, 2016; Visentin et al., 2016). SL biosynthesis mutants *max3* and *max4* and the SL signaling mutant *max2* are more sensitive to drought, due to a relationship between SL and ABA (Bu et al., 2014; Ha et al., 2014). Similarly, osmotic stress had a more severe effect on the *Lotus japonicus* SL biosynthesis mutant: *Ljccd7* (Liu et al., 2015). Nitrogen and phosphorus starvation activated SL biosynthesis and exudation and available reports indicate that SL plays a role in plant adaptation to P deficiency (Umehara et al., 2008; Kohlen et al., 2011; Ruyter-Spira et al., 2011; Andreo-Jimenez et al., 2015). Symbiotic microorganisms including endophytic fungi facilitate adaptation to environmental challenges including nutrient deficiencies and drought (reviewed in Bacon and White, 2016).

Strigolactones are signaling molecules involved in plant-soil microorganism interactions (reviewed in López-Ráez, 2016; Waters et al., 2017). The best described is its action in the plant–AMF interaction. In this mutual relationship the fungus provides the plant with necessary nutrients in exchange for reduced carbon. The AMF symbiosis is widespread throughout the plant kingdom; according to available reports, the roots of over 80% of terrestrial plants are colonized by AMF (Smith and Read, 2008; Brundrett, 2009). Only a few plant families, including the Brassicaceae have lost the ability to engage in mutual symbiosis with AMF (reviewed in Venkateshwaran et al., 2013). Nevertheless, recent reports indicate that numerous members of this family harbor a wide variety of fungal symbionts, including beneficial endophytic fungi that may play a similar role in plant physiology as AMF (Barzanti et al., 2007; García et al., 2013; Card et al., 2015; Hong et al., 2015). This also allows to study the mechanisms of symbiosis with well-established plant models such as *Arabidopsis thaliana*, *Thlaspi caerulescens* etc.

Strigolactones are secreted from plant roots into the rhizosphere and act as a signal for directional growth of the hyphae, thus SL seems to facilitate the plant-AMF interaction in the pre-symbiotic stage of symbiosis (Akiyama et al., 2005; Besserer et al., 2006; Kretzschmar et al., 2012; Mori et al., 2016). Fungal mycelium treated with synthetic SL analogs exhibits a number of changes such as: hyphal branching and growth, increased respiratory activity and ATP and NADPH production, mitosis, expression of effector genes and spore germination. Additionally, SL treatment activates synthesis and release of short chain chitin oligomers which can activate the symbiotic (SYM) signaling pathway, which in turn trigger symbiotic responses in the plant (Lopez-Raez et al., 2017). According to studies with SL-biosynthesis and SL-exudation mutants of pea, petunia, rice and tomato, SL was not necessary for the establishment of the plant-AMF symbiosis, however, the colonization rate of these mutants was much lower compared to wild type plants (Gomez-Roldan et al., 2008; López-Ráez et al., 2008; Vogel et al., 2009; Koltai et al., 2010; Gutjahr et al., 2012; Kretzschmar et al., 2012). The response of different AMF species differs in respect to various SL molecules, however, doses as low as 10 nM of GR24 were shown to affect the growth pattern of the fungi (Besserer et al., 2008). Even though significant progress has been made in elucidating SL signaling and perception in *A. thaliana* and rice, the mechanism of SL perception nor signaling are not known in fungi. In *A. thaliana* the α/β hydrolase D14 was recognized as a SL receptor. SL signaling is mediated by the MAX2 (MORE AXILARY GROWTH2)/SMAXL (SUPRESOR OF MAX2 6, 7, and 8 in particular) signal transduction pathway, but no clear MAX2 or D14 homologs were found in sequenced fungal genomes including *Rhizophagus irregularis* (Waters et al., 2017).

Strigolactones are also involved in other interactions in the rhizosphere: act as a signal for rhizobacteria, as stimulants of parasitic plant seed (*Striga* sp. and *Orobanche* sp.) and pathogenic fungi (Rochange, 2010; Foo and Reid, 2013; Koltai and Kapulnik, 2013). The role of SL in plant–pathogenic fungi interactions is not clear. There are several, contradictory reports. A range of responses to the synthetic SL analog-GR24 on pathogenic fungi growth and branching were reported for the same species (Dor et al., 2011; Torres-Vera et al., 2014; Foo et al., 2016). Recently, Belmondo et al. (2017) has shown that a thioredoxin reductase is necessary for limiting *Botrytis cinerea* growth by GR24, indicating a relationship between SL and ROS (reactive oxygen species) metabolism.

The role of SL in biotic stress responses may be associated with its interaction with other phytohormones or hormone dependent signaling. In the SL deficient tomato, slccd8, reduced concentration of ABA, SA and JA were shown (Torres-Vera et al., 2014). In response to the parasitic plant *Phelipanche ramosa*, the expression of SL biosynthetic D27 and CCD8 and SA, JA and ABA marker genes was upregulated (Torres-Vera et al., 2016). The SL signaling mutant *max2* was more susceptible to *Pectobacterium carotovorum* and *Pseudomonas syringe*, probably due to alterations in ABA metabolism (Piisilä et al., 2015). However, studies performed on SL biosynthesis and signaling garden pea mutants contradict these reports, showing no increased sensitivity to infection by the necrotrophic soilborne oomycete *Pythium irregulare* (Foo et al., 2016).

Previously, the endophytic fungus *Mucor* sp. was found to accelerate *Arabidopsis arenosa* and *A. thaliana* growth (Rozpądek et al., 2017). The fungus was also shown to improve *A. arenosa* toxic metal tolerance (Rozpądek et al., 2017). Other members of this genus improved oilseed rape growth in heavily polluted environments (Zhu et al., 2015; Zahoor et al., 2017) As recently suggested by Martin and Plett (2015), the closely related with AMF Mucoromycetes associated with extant, basal land plants, such as liverworts, hornworts and lycopods, in a symbiosis whose mutualistic nature is suspected, making this group of fungi a good model for studying the mechanisms of symbiosis.

In this study, we evaluated the role of SL in the interaction between *A. thaliana* and its fungal symbiont *Mucor* sp. It was hypothesized that SL is necessary in the development of mutualism between the two interacting organisms both as a secretory signal adjusting the *Mucor* sp. metabolism, to the mutualistic mode and a plant, intrinsic regulatory molecule. Its role was assumed to be associated with its connection to SA synthesis or signaling. Additionally, the possibility that the fungi may have an effect on SL metabolism after colonization was tested.

## Materials and Methods

### Plant Cultivation

*Arabidopsis thaliana* WT (N6000), *max1* (N9564), *max4-1* (N9568) and *max2-2* (N9566) (more axillary branches 1, 4 and 2) mutants (all in Col-0 background) were obtained from NASC (The Nottingham Arabidopsis Stock Centre, United Kingdom). Seeds were surface sterilized with 8% NaOCl, 96% and 75% EtOH and sown to sterile ¼ MS medium in a petri dish and placed in darkness (4°C). After 48 h seeds were transferred to a growth chamber (Panasonic MLR-352H-PE, JP) with a 16 h photoperiod, 21/17°C day/night temperature and 50% humidity. After 10 days seedlings were moved to MSR medium with no sugar (10 plants per petri dish) and inoculated with the fungus. To evaluate the effect of SL on plant biomass yield, MSR was supplemented with 1 μM of the synthetic SL analog: GR24 (StrigoLab, I) in acetone. Inoculation of *in vitro* cultures was performed by placing 2.1 × 10^6^ *Mucor* sp. spores 5 mm from the tip of the main root. After 10–12 days of growth plants were harvested, frozen in liquid nitrogen and stored at -80°C. For biomass yield evaluation 3 separate experiments with 25–30 plants were performed. Due to differences in plant growth in between experiments, fresh weight of treated plants (E+, GR24 and E+GR24) was presented in relation to appropriate control. In all GR24 feeding experiments acetone mock control was performed.

### Strigolactone Feeding Experiments, Fungi Growth, Respiration

*Mucor* sp. (KU234656, strain UNIJAG.PL.E50) spores (2.1 × 10^6^) were placed in PDA (potato dextrose agar) medium supplemented with GR24. For colony growth evaluation spores were inoculated onto PDA containing 1, 10, 50, 100, 500, and 1000 nM of GR24 in 9 cm petri dishes. Colony surface area was measured after 48 h of growth in 24°C in darkness. Fungi respiration was measured with a 30 channel Micro-oxymax respirometer (Columbus Instruments, United States) between the 24 and 48 h of growth. A single O_2_ measurement was performed every 2 h. Spores were placed in PDA supplemented with 50, 500, and 1000 nM of GR24 in 250 ml Duran bottles in the darkness at 24°C (growth chamber of Memmert, IPP400, United States). Fungi respiration was measured as O_2_ consumption per 2 h for 24 h. The respiration rate was presented in relation to colony diameter. The experiment was run in 5 replicates. For all experiments mock (acetone) treated control was performed.

### Enzyme Activity Assays

#### Protein Extraction and Quantification

*Mucor* sp. colonies grown in PDA supplemented with 1, 10, 50, 100, and 1000 nM of GR24 were harvested from media after 48 h of growth and grounded with a mortar and pestle in liquid nitrogen. For crude protein extraction, powdered mycelia were homogenized with molybdenum beads in a TissueLyzer LT (Qiagen, DE) in ice cold 100 mM HEPES-NaOH buffer (pH 7.5, 4 mM DTT, 1 mM EDTA) at 35 Hz for 15 min. The homogenizer adapter was precooled in liquid nitrogen to keep samples frozen. After extraction, samples were centrifuged for 10 min at 10,000 *g* at 4°C. Protein content was quantified according to Bradford (1976) using BSA as a standard. A separate set of fungi mycelium was prepared for each enzyme activity assay. The experiment was run in 5 replicates. For all experiments mock (acetone) treated control was performed.

#### Catalase Activity

The spectrophotometric measurement was performed according to the modified method described by Aebi (1984). Crude tissue extracts (10 μl) were added to 990 μl of phosphate buffer pH 7.0 containing 3 mM H_2_O_2_. CAT activity was determined from the decrease in absorbance at 240 nm due to CAT dependent reduction of H_2_O_2_. Enzyme activity was defined as 1 μmol of H_2_O_2_ decomposed by 1 mg of total soluble proteins per minute.

#### Glutathione Reductase Activity

The supernatants were analyzed for GR activity according to the modified method described by Foyer et al. (1995). Enzyme activity was determined from the decrease in absorbance at 340 nm in the reaction mixture containing TRIS-HCl (50 mM, pH 7.5) buffer, EDTA (1 mM) and GSSG (0.5 mM) in a total volume of 1 ml. Reaction was initiated with the addition of 0.15 mM NADPH.

#### Superoxide Dismutase Activity

Separations of soluble protein fractions were performed using native non-continuous PAGE in the buffer system described by Laemmli (1970) at 4°C and 180 V. SOD bands on 12% polyacrylamide gels were visualized according to the staining procedure described by Beauchamp and Fridovich (1971). The gels were incubated in the staining buffer for 30 min, in darkness, at room temperature and subsequently exposed to white light until SOD activity bands became visible. The gels were scanned using the office scanner Epson V700 Photo, and densitometric analysis was performed with ImageJ (NIH, United States).

### Determination of H_2_O_2_ Concentration

For H_2_O_2_ assay powdered mycelia were homogenized with molybdenum beads in a TissueLyzer LT (Qiagen, DE) in ice cold 50 mM phosphate buffer pH 7.0 and at 40 Hz for 15 min. The homogenizer’s adapter was precooled in liquid nitrogen to keep frozen. After extraction, samples were centrifuged for 15 min at 13,000 *g* at 4°C. H_2_O_2_ was assayed with Amplex^®^ Red Hydrogen Peroxide Kit (Invitrogen) according to the manufacturer’s instructions. The experiment was run in 5 replicates.

### Fungi Staining, Confocal Microscopy and Plant Colonization Assessment

Plant colonization by the fungus was assessed according to Domka et al. (under review), by comparing the expression of the fungal *TEF1α* (Translation elongation factor 1-alpha) with plant *ACT7* (Actin-7) with *q*PCR. To visualize mycelium *in planta* GFP-expressing strain of *Mucor* sp. (KU234656, strain UNIJAG.PL.E50) was generated (Domka et al., under review). Visualization was performed with a confocal microscopy (Nikon Eclipse, JP) equipped with GFP filter blocks.

### SL Decomposition Assay

The ability of the fungi to decompose SL *in planta* was verified by transferring 10 day old seedlings from ccc MS medium to MSR supplemented with 1 μM GR24 fluorescent analog: GR24-BODIPY (StrigoLab, I) for 24 h to allow the plant to uptake it. Subsequently, seedlings were transferred to fresh MSR and inoculated with *Mucor* sp. Plants were harvested after 48 h of growth. Visualization of the fluorescence signal was performed with confocal microscopy (Nikon Eclipse, JP). Fluorescence was excited by 490 nm The fluorescence signal intensity was measured with ImageJ (NIH, United States). Five petri dishes with 10 seedlings for treated and not treated plants were prepared.

### Salicylic Acid and Jasmonic Acid Biosynthesis Induction

To test the relationship between SL and SA and JA production, *A. thaliana* 10 day old seedlings grown in MS medium were transferred to MSR supplemented with 0, 1, 10, 50, 100, 500, 1000 nM of GR24 and harvested after 10 days of vegetation. To evaluate the impact of the fungi on SA production in SL treated plants, *A. thaliana* seedlings were transferred from MS to MSR supplemented with 1 μM GR24 and simultaneously inoculated with *Mucor* sp. (as described in “Plant cultivation”). The temporal pattern of SA production dynamics was evaluated with E+ seedlings grown in medium supplemented with GR24 for 1, 2, 5, and 10 days.

### Salicylic Acid and Jasmonic Acid Concentration Measurement

Salicylic acid concentration was measured 1, 2, 5, and 10 days after transferring seedlings to MSR supplemented with 1 μM GR24. Inoculation was performed simultaneously with transfer. Unlabeled SA was purchased from Sigma-Aldrich (D). Sample preparation and HPLC analysis were carried out according to Müller et al. (2011) with modifications. Frozen plant roots (about 200 mg Fw – 30 plants per sample) was powdered in liquid nitrogen with a metal pestle in polypropylene tubes and then extracted with methanol:isopropanol:glacial acetic acid (20:79:1; v/v/v) in a 10:1 v/w ratio for 20 min in 4°C. During extraction sonification was applied. Subsequently, samples were centrifuged for 20 min in 15000 *g*. This procedure was performed 5 times to assure maximum, close to 100% extraction (from the second extraction 1 ml of extraction solution was used).

The HPLC analysis was performed using Shimadzu LCMS-2020 (JP) system equipped with an autosampler. Separation of plant extracts was performed with a Kinetex 2.6u C18 100x2.1 mm column. The total eluent flow was 0.400 ml min^-1^. Gradient profile described in Müller et al. (2011). The MS analysis was performed using quadrupole mass spectrometer (Shimadzu) negative mode. The following MS parameters were used for analysis: DL temp. 250°C, HB temp. 200°C, detector voltage 0.95 kV, oven temp. 35°C, nebulizing gas flow 15 l min^-1^. The external standard calibration curve method was used for determination of hormone concentration in plant tissues. Five standard solutions were prepared ranging from 0.05 to 10 ng μl^-1^. All samples were run in 3 replicates.

### Gene Expression Analysis

#### RNA Preparation

Total RNA was extracted from frozen in liquid nitrogen, ground leaves (from 5 plants per sample) with the Total RNA Mini Kit (Bio-Rad, United States). RNA purity and quantity was determined by Biospec-Nano (SHIMADZU, JP) The integrity of RNA was assessed with the Agilent 2100 Bioanalyzer (United States) and RNA 6000 Nano Kit (Agilent, DE).

#### *q*PCR

Reverse transcription was carried out on 1000 ng of total RNA, after digestion with DNase (DNA free kit, Ambion Bioscience, United States), with iScript cDNA synthesis kit (Bio-Rad, United States). For *q*PCR, probes were labeled with the EvaGreen (SsoFast EvaGreen Supermix, Bio-Rad, United States) fluorescent dye. For a single reaction 10 ng of cDNA and 150 nM of gene specific primers were used. To test amplification specificity a dissociation curve was acquired by heating samples from 60°C to 95°C. As house-keeping reference α-tubulin 5 (At5g19780) and ubiquitin 10 (At4g05320) was used. Reaction efficiency was tested by serial dilutions of cDNAs with gene specific primers (Supplementary Table 1). All samples were run in triplicates. Expression was calculated according to Pfaffl (2001) with WT plants serving as calibrator. For gene expression analysis plants were harvested 10–12 days after inoculation. The experiment was repeated twice. In each experiment 3 samples (10 plants per sample) per variant were collected. Analysis were performed in triplicates.

### Statistical Analysis

Data normality and variance homogeneity were evaluated by the Shapiro–Wilk and Levene’s tests, respectively. If necessary, data were normalized with log or Box-Cox transformation. Statistical significance was determined by analysis of variance (ANOVA), followed by Tuckey or Fischer *post hoc* test (*p* ≤ 0.05) as indicated in figure captions. Differences between two groups were tested by *t*-test. Statistical analyses were conducted using Statistica ver. 12.5 (Statsoft).

## Results

### GR24 Inhibits Mycelium Growth and Increases the Respiration Rate

**FIGURE 1:**
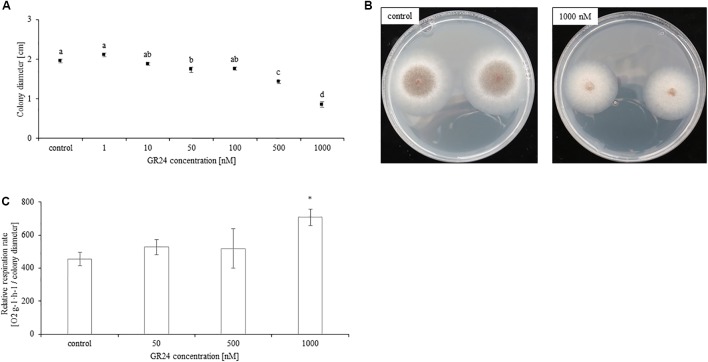
*Mucor* sp. colony diameter measured 48 h after PDA inoculation with 2.1 × 10^6^ fungi spores. The medium was supplemented with 0, 1, 10, 50, 100, 500, and 1000 nM of GR24 **(A)**. Letters above bars indicate statistically significant differences according to one-way ANOVA and Tuckey *post hoc* test (*N* = 10, *P* ≤ 0.05). Respiration rate of Mucor sp. grown in PDA supplemented with 50, 500, and 1000 nM of GR24 and not treated control. Fungi respiration was measured as O_2_ consumption per 2 h for 24 h. The respiration rate was presented in relation to colony diameter. Stars above bars represent statistically significant differences according to the student’s *t*-test (*N* = 5, *P* ≤ 0.05, mean value ± SE) **(B)**. Photographs illustrating *Mucor* sp. colony diameter grown for 48 h in PDA supplemented with 1000 nM GR24 **(C)**.

Supplementation of growth medium with GR24 significantly inhibited the growth of *Mucor* sp. Colony diameter decreased with growing concentrations of GR24. The lowest concentration which had an inhibitory effect was 50 nM. Treatment with 100 nM inhibited mycelium growth in a similar fashion, whereas treatment with concentrations of 500 and 1000 nM resulted in a gradual decline in colony diameter (**Figures 1A,B**). Additionally, as shown in **Figure 1B** treatment with 1000 nM delayed spore formation by the fungi. The respiration rate was significantly decreased only in result of treatment with 1000 nM of GR24. Lower concentrations did not significantly affect it (**Figure 1C**). Mock treatments did not differ from control.

### GR24 Activates H_2_O_2_ Production and Alters the Activity of Antioxidant Enzymes

**FIGURE 2:**
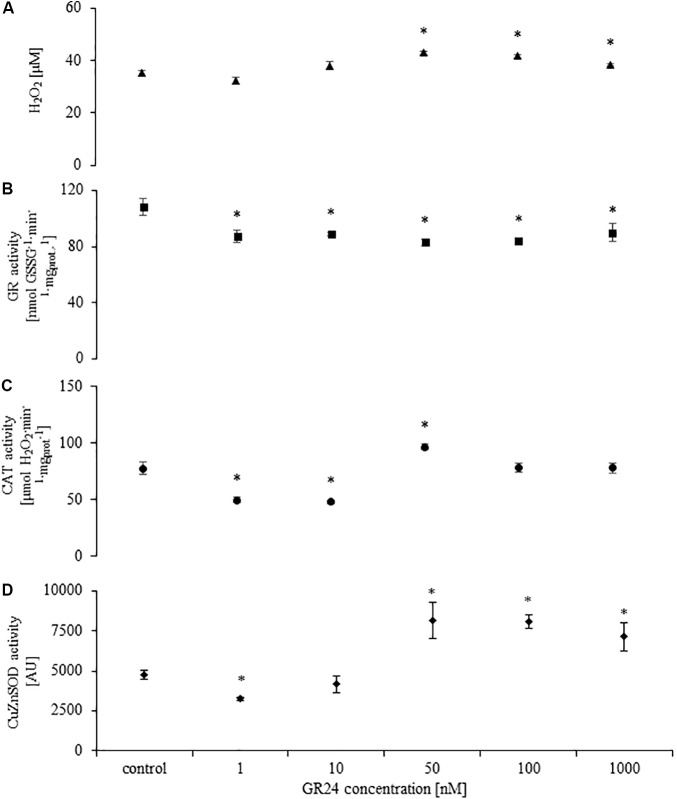
H_2_O_2_ concentration **(A)**, glutathione reductase (GR; **B**), catalase (CAT; **C**), superoxide dismutase (Cu/ZnSOD; **D**) activities in *Mucor* sp. treated with 0, 1, 10, 50, 100, and 1000 nM of GR24. Stars above bars represent statistically significant differences according to the student’s *t*-test *N* = 5, *P* ≤ 0.05, mean value ± SE.

GR24 treatment with doses higher than 50 nM induced H_2_O_2_ production in the fungi mycelium. There were no difference in H_2_O_2_ concentration in mycelium treated with 50, 100, and 1000 nM (**Figure 2A**) The activity of GR was significantly decreased in all treatments (**Figure 2B**). The response of CAT and Cu/ZnSOD was dose dependent. Doses of 1 and 10 nM decreased enzyme activity (**Figures 2C,D**) whereas 10–1000 nM significantly increased Cu/ZnSOD, but not CAT activity (**Figures 2C,D**). Mock treatments did not differ from control.

### Fungi Colonization of SL Biosynthesis Mutants

**FIGURE 3:**
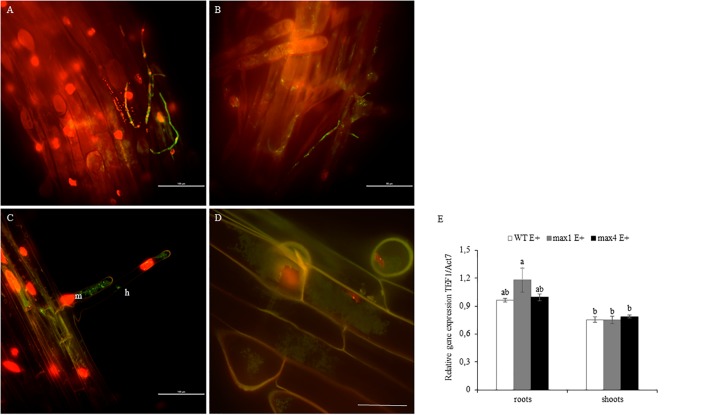
Roots of *A. thaliana* colonized by *Mucor* sp. tagged with GFP. Fungal hyphae on the root surface, bars 100 and 50 μm respectively **(A,B)**. Mycelium (m) inside root hair (h), bar 100 μm **(C)**. Fungal mycelium (m) inside root cells, bar 50 μm **(D)**. Root colonization rate by mycelium shown as fungal TEF1α gene expression in relation to plant ACT7 gene expression. Letters above bars indicate statistically significant differences according to one-way ANOVA and Tuckey *post hoc* test (*N* = 3, *P* ≤ 0.05, mean value ± SE) **(E)**.

One day after inoculation fungal hyphae was detected on the surface of the root (**Figures 3A,B**). Later, the mycelium was present inside root hairs (**Figure 3C**) and inside other root cells (**Figure 3D**). Colonization of *A. thaliana* tissues by *Mucor* sp. was described in detail by Domka et al. (2017, submitted). No significant changes in colonization rate were observed (**Figure 3E**).

### SL Biosynthesis Mutants Do Not Develop the Beneficial Growth Phenotype Upon Inoculation

**FIGURE 4:**
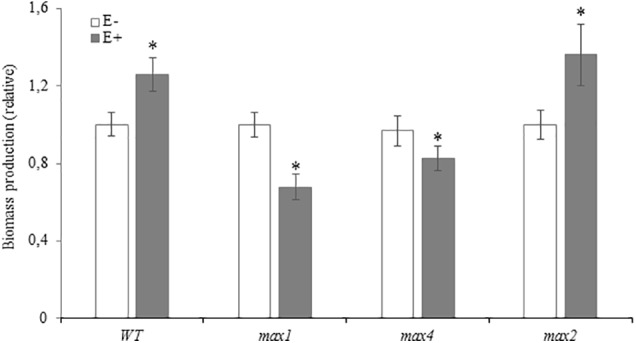
Fresh weight of *Arabidopsis thaliana* WT and max1, max4 and max2 mutants 10 days after inoculation with *Mucor* sp., GR24 treated and inoculated with *Mucor* sp. and treated with GR24 **(A)**. Three separate experiments with 25–30 plants per genotype were performed. Due to differences in plant growth in between experiments, fresh weight of treated plants (E+, GR24 and E+GR24) was presented in relation to appropriate control (WT, max1, max4, max2). Stars above bars represent statistically significant differences according to the student’s *t*-test *N* = 5, *P* ≤ 0.05, mean value ± SE.

Due to differences in growth tempo between experiments, bars in **Figure 4** represent fresh weight treated plants relative to appropriate control: WT, *max1*, *max4* and *max2*. Inoculation with *Mucor* sp. improved biomass yield of WT and *max2 A. thaliana*, whereas SL biosynthesis mutants produced significantly less biomass (**Figure 4**).

### *Mucor* sp. Decomposes GR24 *in Planta*

**FIGURE 5:**
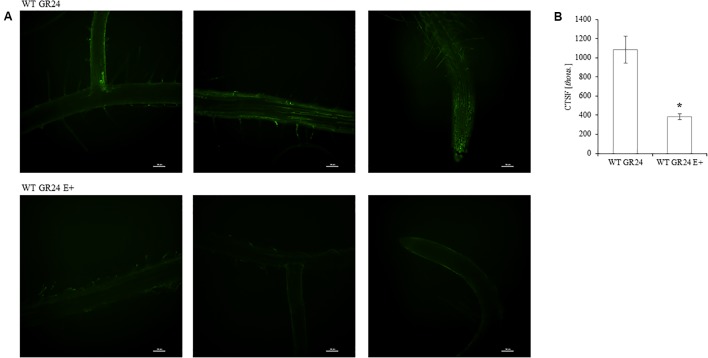
Photographs illustrating the decomposition of the fluorescent analog GR24-BODIPY (StrigoLab, I) by *Mucor* mycelium inside roots of *A. thaliana*. Plants were fed with 1 μM GR24-BODIPY in MSR for 24 h transferred to GR24 free medium and inoculated with *Mucor* sp. **(A)**. Visualization was performed with confocal microscope and quantification of the fluorescence signal was performed 48 hpi with ImageJ-NIH. Stars above bars represent statistically significant differences according to the student’s *t*-test *N* = 3, *P* ≤ 0.05, mean value ± SE **(B)**.

The fluorescence signal from GR24-BODIPY was significantly lower in E+ plants 48 h after inoculation (**Figures 5A,B**).

### High Doses of GR24 Induce SA Synthesis in *A. thaliana*, But Has No Effect on JA Production

**FIGURE 6:**
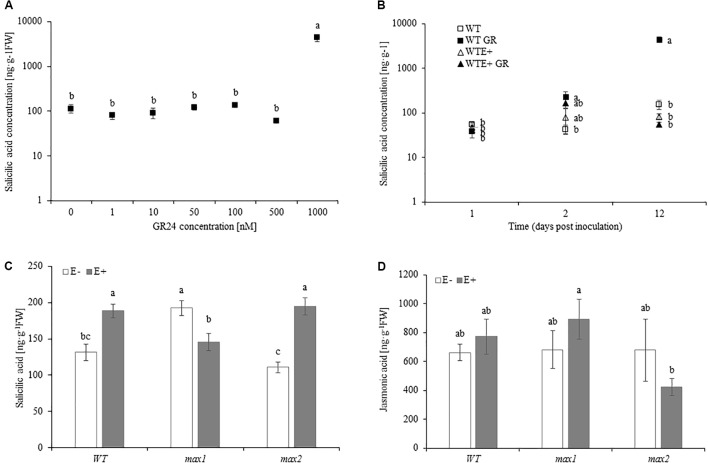
SA accumulation in *Arabidopsis thaliana* WT grown in medium supplemented with 0–1000 nM of GR24 for 10 days **(A)**. SA accumulation in *Arabidopsis thaliana* WT grown in medium supplemented with 1000 nM of GR24 for 24 h, 48 h and 12 days **(B)**. SA **(C)** and JA **(D)** concentration in *A. thaliana* seedlings inoculated with *Mucor* sp. 10 days after inoculation. Letters above bars indicate statistically significant differences according to one-way ANOVA and Fischer *post hoc* test (*N* = 3, *P* ≤ 0.05, mean value ± SE).

To test the relationship between SL and SA and JA production, *A. thaliana* 10 day old seedlings grown in medium MS medium were transferred to MSR supplemented with 0, 1, 10, 50, 100, 500, 1000 nM of GR24. Synthetic SL treatment had no effect on JA accumulation in plants (data not shown). SA concentration was significantly increased (close to 30-fold) in plants treated with the highest dose of GR24 (**Figure 6A**). Lower concentrations did not affect SA synthesis in *A. thaliana.* No differences in SA concentration were shown 24 hpi (hours past inoculation) (**Figure 6B**). 48 hpi GR24 treated seedlings accumulated significantly more SA. Twelve days after inoculation GR24 treated plants accumulated 25-fold more SA compared to control. Inoculation with the fungi prevented SA production by the plant (**Figure 6B**). 48 hpi SA accumulation was slightly lower than in untreated (GR24+) plants, but not significantly higher than in control and E+ seedlings. No differences in SA concentration were shown 12 dpi between control and E+ seedlings. Mock treatments did not differ from control.

### Salicylic Acid and Jasmonic Acid Accumulation in E+ WT and *max1* and *2* Mutants

SA accumulation in *max1* mutants was significantly higher, whereas *max2* accumulated significantly less SA than in WT plants. Upon inoculation SA concentration increased in WT and *max2*. *max1* mutants responded to inoculation with decreased SA accumulation, which was significantly lower than in WT and *max2* (**Figure 6C**). No differences in JA accumulation were shown between WT, *max1* and *max2* mutants, nor did inoculation affect it (**Figure 6D**).

### The Expression SL Biosynthesis Was Downregulated in E+ *Arabidopsis thaliana*

**FIGURE 7:**
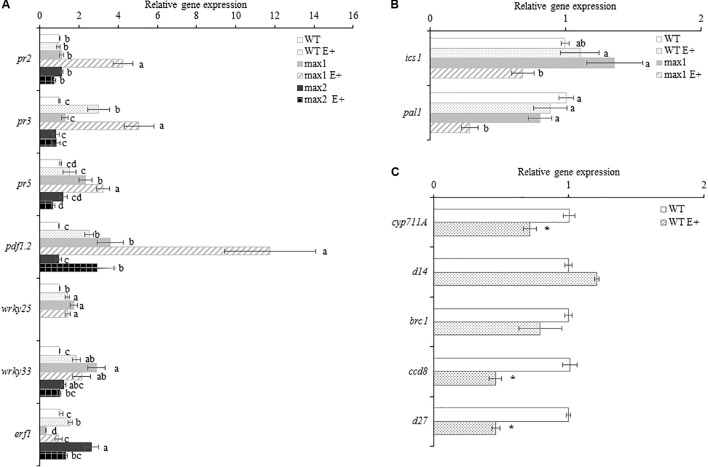
The expression of *A. thaliana* WT, *max1* and *max2* inoculated with *Mucor sp.* (E+) defense related genes **(A)**, salicylic acid biosynthesis genes **(B)** and strigolactone biosynthesis and signaling genes **(C)** Letters above bars indicate statistically significant differences according to one-way ANOVA and Fischer *post hoc* test (*N* = 6, *P* ≤ 0.05, mean value ± SE).

The expression of genes encoding proteins involved in SL biosynthesis: *CYP711A* (cytochrome P450, family 711, subfamily A), *D27* (beta-carotene isomerase D27-like protein) and *CCD8* (carotenoid cleavage dioxygenase 8) were significantly downregulated in inoculated WT plants, whereas genes encoding proteins involved in SL signaling were either unaffected by inoculation: *D14* (strigolactone esterase D14), *BRC1* (branched 1) (**Figure 7C**).

### The Expression Profiles of Plant Defense Related Genes Were Altered in *max1* and *max2* Mutants During the Interaction With *Mucor* sp.

In order to evaluate the role of SL in adjusting *A. thaliana’s* defense to inoculation with *Mucor* sp. the expression of selected plant defensins was quantified (**Figure 7A**). Upon inoculation, the abundance of *PR2* mRNA was not changed in WT and *max2*. *max1* exhibited a significant upregulation of the expression of this gene. The expression of PR3 was upregulated in E+WT and E+*max1* (in *max1* more significantly then in WT), whereas in *max2* no differences in PR3 expression after inoculation were shown. No differences in the expression of PR5 between E+ and appropriate controls were shown, however, in the SL biosynthesis mutant PR5 transcript abundance was significantly higher compared to WT and *max2* (in both control and E+). Interestingly, PR5 expression in E+*max4* was significantly higher (Supplementary Figure 1).

Inoculation with *Mucor* sp. resulted in a significant upregulation in the expression of PDF1.2 in relation to relevant controls in all plants examined. Expression levels in WT and *max2* were similar, whereas PDF1.2 transcript abundance in *max1* was significantly higher (in both control and E+ plants). The expression of two defense related transcription factors WRKY25 and WRKY33 was upregulated in *max1* compared to WT plants. After inoculation their abundance rose in WT, whereas in *max1* and *max2* no differences were found. The response of SA biosynthesis genes was also altered in SL biosynthesis mutants. Genes encoding ICS1 and PAL1 were downregulated upon inoculation in *max1* E+ in relation to respective control, whereas no differences in their expression were found in WT (**Figure 7B**). The expression of the examined genes in *max4* closely resembled that in *max1* (Supplementary Figure 1).

## Disscusion

GR24 strongly inhibited the growth of a number of phyto-pathogenic fungi, suggesting a role in plant defense (Dor et al., 2011). Additionally, hyphal branching was shown to be activated upon treatment with the SL analog. In this study GR24 treatment had a similar effect on the endophytes growth, but it did not affect branching (data not shown). GR24 doses used in this study were, however significantly lower compared to the concentrations used by other authors. Previously, micromolar concentrations of GR24 were shown to limit growth of mycelium (Dor et al., 2011; Belmondo et al., 2017), whereas here, 50 nM of GR24 was sufficient enough to limit colony expansion. This suggests that SL in the mutualistic interaction, imposes its effects in concentrations relatively lower compared to plant-pathogen interactions. Higher pathogen resistance to SL may have evolved due to selective pressure of this group of fungi. This, however, requires more detailed research.

Mycelium growth inhibition was accompanied by increased production/accumulation of H_2_O_2_ and inhibited the activity of GR which plays an important role in H_2_O_2_ scavenging. Lower concentrations of GR24, below 50 nM, reduced the activity of antioxidant enzymes examined, indicating that SL activates ROS production in the mycelium either directly by inducing an oxidative burst or indirectly, by reducing the activity of ROS scavenging enzymes. ROS were suggested to be necessary in limiting *B. cinerea* radial growth by GR24 (Belmondo et al., 2017). Previously, Tanaka et al. (2006) showed that by knocking out the *NOXA* gene encoding the plasmalemma bound, 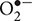 generating NADPH oxidase, the perennial ryegrass endophyte *Epichloë festucae* spreads throughout the plant causing disease symptoms. This indicated that by inducing ROS production the host plant may control its symbiotic partner. GR24 concentrations of 50 nM and above induced the production of H_2_O_2_ in *Mucor* sp. and activated the H_2_O_2_ producing/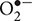 scavenging Cu/Zn SOD, suggesting that SL plays a role in activating ROS production during plant–fungi symbiosis.

Strigolactone are perceived by D14, a non-canonical α/β hydrolase receptor. Upon binding D14 was proposed to hydrolyse SL (Koltai and Kapulnik, 2013). The mechanism of SL perception by fungi is unknown, however, SL decomposition is not a unique plant feature. Recently it was shown that soil borne fungi from the *Trichoderma* and *Fusarium* genus were able to degrade 4 different natural and synthetic SLs including GR24. This ability is considered to be used in prevention of parasitic plant seed germination, but the biological relevance of this phenomenon is not known (Boari et al., 2016). In this study, we showed that *Mucor* sp. can also decompose GR24, both *ex planta* and more importantly *in planta*, thus it cannot be excluded that the fungi can modulate SL metabolism during the colonization process. Additionally, the results presented here show that upon inoculation, the expression of SL biosynthesis genes was downregulated in *A. thaliana*. This provides further evidence for the modulatory role of the endophytic *Mucor* sp. on SL metabolism during symbiosis establishment.

To evaluate the relevance of SL in the interaction between *A. thaliana* and *Mucor* sp. SL mutants were inoculated with the fungi. Biosynthesis mutants *max1* and *max4* responded negatively, in terms of growth to inoculation. The phenotype of *max2* (which synthesizes, but does not respond to SL) after inoculation with *Mucor* sp. resembled WT, suggesting that SL may had induced changes in *Mucor* sp. metabolism by switching it from “beneficial to parasitic mode” and MAX2 dependent signaling was not necessary for the development of the beneficial phenotype. What’s interesting is that the colonization rate of *max1* and *4* mutants did not differ from WT, indicating that in the absence of SL the plants did not lose ability to control colonization.

Plants control mycelium spread inside their tissues by activating a specific immune response. Selected defense mechanisms controlled by plant hormones JA, SA and ET are upregulated and others are downregulated (Jacobs et al., 2011; Lahrmann et al., 2015). A relationship between SL and these phytohormones has also been suggested (Marzec, 2016). According to the only report connecting SL to plant defense (Torres-Vera et al., 2014), SL deficiency alters defense related hormone profiles: the *Slccd8* tomato RNAi line accumulated less JA and SA. According to results presented here JA accumulation was not affected by neither SL deficiency, lack of MAX2 dependent SL signaling nor inoculation. To verify weather SL acts in controlling plant defense (possibly in the suppression of plant defense) we quantified the accumulation of SA in WT and *max* mutants inoculated with the fungus. The results indicate that, SL may act in suppressing SA production: *A. thaliana max1* and *max4* (see Supplement) accumulated significantly more SA than WT plants. At the same time, SA concentration in WT E+ and *max2* E+ increased what was coincident with deactivation of SL biosynthesis genes, suggesting that there may be a relationship between these two processes. SA biosynthesis gene expression was not changed in WT plants what indicates that other routes of SA accumulation regulation (SA catabolism for instance) had to control this process (Lahrmann et al., 2015). Exogenously applied synthetic SL activated SA synthesis in *A. thaliana* but only in concentrations of 1 μM, lower concentrations did not affect SA production in seedlings. This undermines the hypothesis of the downregulating role of SL in SA production. The physiological relevance of SL activated SA production is controversial. There are no reports indicating that plants can be exposed to such high concentrations in nature, nM and pM concentrations are usually found *in planta* (Seto et al., 2014), but 1 μM of GR24 is preferentially used in most studies using this SL analog (Marquez-Garcia et al., 2014; Passaia et al., 2014; Ito et al., 2015). In literature, there is only one report indicating that micromolar doses of GR24 can be toxic to the plant (Ito et al., 2015). No reports consider the fact that *A. thaliana* seedlings treated with GR24 can be exposed to stress (SA biosynthesis is stress responsive), nor consider the fact of SA synthesis activation upon GR24 treatment. Nevertheless, the results of SA accumulation seem to confirm that SL serves in inhibition of SA accumulation. SA accumulated in *max1* mutants, co-cultivation of *A. thaliana* with *Mucor* sp. resulted in downregulation of SL biosynthesis genes and an increase in accumulation of SA (probably *via* a *max2* independent pathway). Additionally, we have shown that the fungus has the ability to degrade SL *in planta* what can be another routes of regulating SL content by the fungus. Several reports indicate that mycorrhizal plants prevent further root colonization by AMF possibly by altering SL production (reviewed in Steinkellner et al., 2007). The results presented here provide indirect evidence confirming that, indeed the endophytic fungus *Mucor sp.* possesses the ability to modulate SL metabolism/abundance *in planta*. Verifying the ecological significance of this phenomenon requires, however, further research. The relationship and the role of SL and SA in the establishment of the plant–fungi symbiosis is much more complicated than the picture presented above, the accumulation of SA in *max1* E+ and *max4* E+ mutants, as well as SA biosynthesis gene downregulation in E+ mutants indicates that other factors are involved in establishing the post infection equilibrium between SA and SL in plant–endophytic fungi interactions.

To further explore the relationship between SL and plant defense during the interaction between *A. thaliana* and *Mucor* sp. the expression of selected defense related genes in *A. thaliana* SL was measured. The expression of plant defensins is SA and JA inducible (Thomma et al., 1998, 1999). The expression of the tested defensins (PR2, PR3, PR5, PDF1.2) was significantly upregulated in inoculated plants under SL deficiency. Additionally, *PDF1.2* and *PR5* expression was significantly higher in the absence of the fungal stimulus providing further evidence for a link between SL and plant defense. The expression of the two defense related TFs examined also differed in WT and *max1*. WRKY25 and WRKY33 transcript abundance was higher in *max1* and did not respond to inoculation, whereas WT exhibited an activation of their expression. Surprisingly, the response of the SL signaling mutant *max2* differed from both WT and *max1*. Constitutive expression of the genes tested resembled WT, but, barely any of the genes responded to the fungi. Out of the tested genes only *PDF1.2* exhibited a response similar to that found in WT. Nevertheless, inhibition of *max2* dependent SL signaling resulted in almost complete non-responsiveness of the tested genes. The *max2* mutant produces SL and developed the beneficial phenotype upon inoculation, what indicates that SL serves both as a signal influencing the fungi and a signaling molecule controlling plant defense in response to the endophyte. It seems as though, SL MAX2 dependent signaling and activation of defense that takes place in the WT is not a necessary condition in the development of a mutual symbiosis between *A. thaliana* and *Mucor* sp. However, defense related gene expression in *max1* and *max4* mutants indicates that SL plays a role in suppressing the expression of the tested genes. The MAX2 dependent SL signaling pathway is intact in these mutants, suggesting that other factors compensate for SL under SL deficiency. Additionally, MAX2 signaling is not a unique feature of SL signal transduction. Another group of plant signaling molecules: karrikins, derived from cellulose combustion and involved in fire follower plant seed germination (among other functions) utilize MAX2 dependent signaling (Waters et al., 2014). MAX2 also targets several other proteins including the brassinosteroid target protein BRI1-EMS SUPPRESSOR1 (BES1) and the proteins from the DELLA family of GRAS transcriptional regulators involved in gibberellin signaling (Wang et al., 2013). Thus, caution needs to be taken when interpreting the response of the MAX2 mutant.

During the *A. thaliana*–*Mucor* sp. interaction an accumulation of defense compounds took place in WT (*PDF1.2* and *PR3*), but in significantly less quantities when compared to *max1* and *max4*. Previously, Jacobs et al. (2011) have shown that some basal defense is necessary for the establishment of the beneficial interaction between an endophytic fungus and its host plant. The results presented here seem to confirm this statement, however, there are variations in the response of *A. thaliana* to *Mucor* sp. and other fungi species in terms of activation specific defense related genes.

Upregulated defense mechanisms may not necessarily indicate a direct link between SL and defense. We can also imagine that since SL affects the fungi limiting its growth, it can also affect some unknown recognition patterns rendering the fungi recognizable as an endophyte and the upregulation of plant defense results from not recognizing the fungi as potentially beneficial. Additionally, the negative growth response of SL biosynthesis mutants suggests that in the absence of SL, a shift in resource allocation from growth to defense necessary to restrain fungi spread may take place. This, however, requires further investigations.

## Conclusion

Recently, intense efforts are made to define the elements that allow plants to distinguish between potential symbionts and pathogens and to elucidate the plant-fungi cross-talk. Studying signals exchanged in the rhizosphere by plants and fungi is thus significantly important (Bonfante and Genre, 2010; Antolín-Llovera et al., 2014; Hayashi and Parniske, 2014). The role of SL in this process has been proposed previously. The data presented in this study indicates that SL plays a dual role in the interaction between plants and endophytic fungal symbionts. The synthetic SL analog GR24 was shown to affect the metabolism of *Mucor* sp. by limiting its growth and inducing ROS production. On the other hand, the fungus was shown to be able to decompose SL *in planta* and reduce SL biosynthesis gene expression. At the same time SL deficient *A. thaliana* mutants, but not the *max2* signaling mutant were not able to benefit from its fungal symbiont. The expression of a number of defense related genes was upregulated in SL deficient plants, suggesting a role of SL in regulating the plants immune response. However, it seems that the postulated link between SL and JA, SA is not as unequivocal as previously thought. Nevertheless, we present several lines of evidence of a direct and/or indirect relation of SL and plant defense. Additionally, we have shown that high doses of GR24 activate SA production in *A. thaliana* and that by inoculating the plant with *Mucor* sp. we were able to prevent SA accumulation most probably by limiting root exposition to GR24 (the fungi decomposed GR24). This model represents a good illustration of the role that fungi play in natural environments. SL decomposition and possible downregulation of SL biosynthesis in the host plant limits SL availability in the soil, what in turn may have a limiting effect on parasitic plant seed germination.

## Author Contributions

PR designed the study, participated in experiments, analyzed the data and prepared the text of the manuscript. AD performed the *q*PCR, microscopic analyses. RW performed the *q*PCR and statistical analysis of data. RJ performed the HPLC determination of hormones. MN performed enzyme activity assays. KT was involved in designing the study and interpretation of the results. All authors were involved in the manuscript preparation and approved the manuscript prior to the submission.

## Conflict of Interest Statement

The authors declare that the research was conducted in the absence of any commercial or financial relationships that could be construed as a potential conflict of interest.
