# Supplementary material for: The Role of Strigolactone in the Cross-Talk Between Arabidopsis thaliana and the Endophytic Fungus Mucor sp
Source: Front Microbiol. 2018 Mar 19;9:441. doi: 10.3389/fmicb.2018.00441 (PMC5867299; doi:10.3389/fmicb.2018.00441)
Supplement: Supplementary file 1 [file Data_Sheet_1.docx]

Supplementary Material

**The role of strigolactone in the cross-talk between *Arabidopsis thaliana* and the endophytic fungi *Mucor sp.***

**Piotr Rozpądek^1*^, Agnieszka Domka^2^, Michał Nosek^3^, Rafał Ważny^1^, Roman J. Jędrzejczyk^1^, Monika Wiciarz^4^, Katarzyna Turnau^2^**

*Correspondence: Piotr Rozpądek: piotr.rozpadek@uj.edu.pl

# Supplementary Figures and Tables

## Supplementary Figures

**Supplementary Figure 1.** The expression of *A. thaliana* WT and *max4* inoculated with *Mucor sp.* (E+) defense related genes. Letters above bars indicate statistically significant differences according to one-way ANOVA and Fischer post-hoc test (N=6, P≤0.05).

## Supplementary Table

**Supplementary Table 1.** List of primers used for gene expression

| Name | Accession number | Sequence (5’ – 3’) |
| --- | --- | --- |
| PR1 - pathogenesis-related protein 1 | At2g14610 | F: GTGGGTTAGCGAGAAGGCTA  R: ACTTTGGCACATCCGAGTCT |
| PR2 - pathogenesis-related protein 2 | At3g57260 | F: CCGACAAGTGGGTTCAAGAA  R: CGTATCAGTGGTGGTGTCAG |
| PR3 - pathogenesis-related protein 3 | At3g12500 | F: GCACCAGACGGACCATATTC  R: GCAACAAGGTCAGGGTTGTT |
| PR5 - pathogenesis-related protein 5 | At1g75040 | F: AATTGCCCTACCACCGTCTG  R: AATTCAGCCAGAGTGACGGG |
| PDF1.2 - ethylene- and jasmonate-responsive plant defensin | At5g44420 | F: TCTTCGCTGCTCTTGTTCTC  R: CTTGTGTGCTGGGAAGACAT |
| WRKY25 - WRKY DNA-binding protein 25 | At2g30250 | F: GAACCGGGTCTGGTTTACCT  R: GGAAACGTTCCTGTTGTTGG |
| WRKY33 - WRKY DNA-binding protein 33 | At2g38470 | F: TCACACGACATGAGAGCAGT  R: GAGTGACCAGCAATAGCAGC |
| ICS1 - isochorismate synthase 1 | At1g74710 | F: ATGAGATTCAGCCTCGCTGT  R: TGATGGATCTCCAATCGTCA |
| PAL1 - phenylalanine ammonia-lyase 1 | At2g37040 | F: TGGATTCAAGGGAGCTGAGA  R: TCAGAAGTTTTGCGAGACGA |
| ERF1 - ethylene-responsive transcription factor 1 | At3g23240 | F: GTCCTCGGCGATTCTCAATT R: GAGCGGTGATCAAAGTCACT |
| EDS1 - enhanced disease susceptibility 1 | At3g48090 | F: CCTCGTTGTGTGACATTTGG  R: AATTGGGCAAGAACATGAGG |
| SMXL6 - protein SMAX1-LIKE 6 | At1g07200 | F: CAGACGCTCTCTAGATGCCA  R: ACTGCTGCCTGTTATTCCCT |
| BRC1 - branched 1 | At3g18550 | F: GATCGCGACAACCCTTTCTC  R: TCCCTCTCATCGATCTCCCA |
| D27 - beta-carotene isomerase D27-like protein | At1g03055 | F: AAGACGCAGCTGGGATTAGT  R: TCTGAAGGTCCAACAAGCCA |
| CCD8 - carotenoid cleavage dioxygenase 8 | At4g32810 | F: GTCGACCATGAGACGCTAGA  R: CCATCCTCACGACCCGATAA |
| CYP711A - cytochrome P450, family 711, subfamily A | At2g26170 | F: CCAAGGAGGCTGAGACTGAC  R: TGAAAGCAGTGGTTGCAGAG |
| D14 - strigolactone esterase D14 | At3g03990 | F: CGGAATCCAAAACTGTGCTT  R: GAACATCAGCTCCAACAGCA |
| DLK2 - alpha/beta-Hydrolases superfamily protein | At3g24420 | F: AGAAGATGAAGCCCGAGACA  R: CCACTGTCGATTTCCCCTTA |
| TUA5 – tubulin alpha-5 | At5g19780 | F: CGCCTTCATTTCCTCGATCT  R: GTATTGAACGCATCGTGTGC |
| UBQ10 – polyubiquitin 10 | At4g05320 | F: ATCTTTGCCGGAAAACAATTGGAGGATGGT  R: GACTTGTCATTAGAAAGAAAGAGATAACAGG |
